# Behaviour artificial intelligence technology to support the decision-making process of continuation or withdrawal of life-sustaining therapy

**DOI:** 10.1186/s40635-026-00930-4

**Published:** 2026-07-01

**Authors:** Patrick J. Thoral, Jesse de Metz, Stella Mulia, Annebel ten Broeke, Nicolaas Heyning, Birkitt L. ten Tusscher, Rolf K. Gigengack, Hilde M. Feijen, Caspar G. Chorus, Bas van den Bogaard, Paul W. G. Elbers

**Affiliations:** 1 Department of Intensive Care Medicine, Center for Critical Care Computational Intelligence, Amsterdam Medical Data Science, Amsterdam UMC, Vrije Universiteit Amsterdam, Amsterdam, The Netherlands; 2 Department of Intensive Care, OLVG, Amsterdam, The Netherlands; 3 Councyl B.V., Delft, The Netherlands; 4 Faculty of Industrial Design Engineering, Delft University of Technology, Delft, The Netherlands

**Keywords:** Behaviour artificial intelligence, Expert systems, Decision support, End of life care, Treatment withdrawal

## Abstract

**Background:**

Withholding and withdrawing life-sustaining therapy (LST) is common in European ICUs but significant variations exist. Behaviour artificial intelligence technology (BAIT) may help standardize the ethical dilemma to continue or withdraw LST for patients already admitted to the ICU.

**Methods:**

Several sessions with intensivists of an academic medical centre and a large urban teaching hospital were held to determine the criteria influencing the process. A discrete choice experiment was conducted during which 25 hypothetical cases were presented to the participants. For each case the participants had to decide whether they would continue, continue with a time limited trial of one week, or withdraw LST. The results of the experiment were used to develop a multinomial logistic regression model that was incorporated in a web-based decision-support system.

**Results:**

Thirty-six participants (intensivists and fellows in intensive care medicine) completed the experiment. The estimated model consisted of twelve covariates and showed good model fit (McFadden’s *ρ*^2^ 0.25). The most important covariates were age, patient values, expected cardiovascular and pulmonary impairment after ICU discharge and frailty at admission. The BAIT system lets intensivists view expected decisions based on documented criteria and uses color-coding to show the magnitude of the effect and its direction (i.e. to continue or withdraw LST).

**Conclusions:**

We developed a BAIT system that may support clinicians facing the dilemma of continuing or withdrawing LST by elucidating the key criteria involved in assessing medical futility.

**Supplementary Information:**

The online version contains supplementary material available at 10.1186/s40635-026-00930-4.

## Background

The progress of medicine, and intensive care medicine in particular, has allowed the application of life-sustaining therapy (LST) to treat potentially reversible conditions that would otherwise have been fatal. However, starting or continuing intensive treatment in every situation may not be in the best interest of each individual patient and society as well due to the high costs and resource usage associated with LST [1].

Current guidelines for example suggest against initiation of cardiopulmonary resuscitation if it is obviously futile [2], but futility varies between sociocultural and personal beliefs and is also influenced by legal implications [3–5].

Both withholding and withdrawing intensive care treatment is common in European ICUs but significant regional variations exist [6, 7]. In the Netherlands, both withholding and withdrawing are legally permitted when treatment is considered futile by the practicing physician. Since there is no uniform definition of futility of medical treatment, many guidelines suggest that decisions relating to withholding or withdrawing treatment should be taken in interdisciplinary meetings and as part of shared decision-making during family conferences to elicit patients’ values and goals [8, 9].

However, the organization of intensive care units vary and the composition of interdisciplinary meetings and the interactions between team members during these meetings may vary [10]. Consequently, the evaluation of medical futility may also vary.

Behavioural artificial intelligence technology (BAIT), a form of discrete choice analysis, is a technique that allows capturing the expertise of domain experts (e.g. intensivists) by analysis of a choice experiment [11]. Choice analysis techniques have traditionally been used to predict future choices by identifying the preferences of large groups of consumers, citizens, or patients. While originally an econometric technique, models have been successfully reconceptualized as decision support or expert systems and applied in the intensive care domain to support admitting COVID-19 patients [12] and performing surgery on neonates with necrotizing enterocolitis [11].

While a number of authors have proposed to use computerized decision support to aid the difficult ethical dilemmas surrounding withdrawal of LST [13–15], the number of models that could support this decision is currently scarce [16].

In this work we evaluated whether behavioural artificial intelligence could capture the criteria that determine the decision to continue or withdraw LST. In addition, we aimed to develop a decision support system to ultimately aid this dilemma.

## Methods

### Situation

Intensivists and fellows in intensive care medicine of a tertiary academic medical centre (Amsterdam UMC, AUMC) and a large teaching hospital (OLVG) both situated in Amsterdam, The Netherlands were invited to participate in the experiment.

### Design

**Fig. 1:**
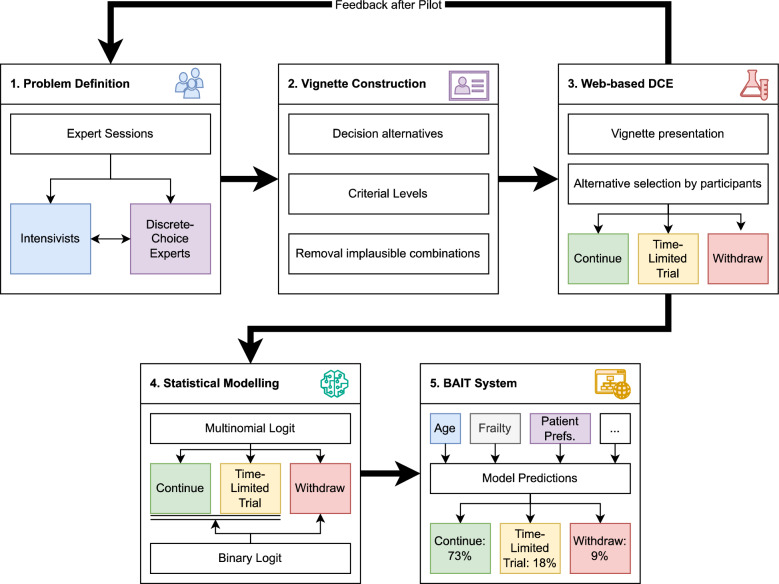
Study design. Schematic showing the five steps of developing the Behavioural Artificial Intelligence Technology (BAIT) system. 1. During Expert sessions the clinical decision problem is defined, including criteria influencing that decision. 2. Based on the input of the expert sessions, vignettes are constructed, using specified criteria levels and removing clinically implausible combinations. Using the ChoiceDesign software efficient combinations of criteria are chosen to create the vignettes. 3. The vignettes are presented to participants in a web-based discrete choice experiment (DCE). In the experiment, participants are requested to document their decision (choice) based on the presented vignette. 4. Using the results of the DCE, statistical models are developed using Apollo software. 5. The statistical models are used in the BAIT system, a web-based system, to show predictions based on the input of the predictors. In the current study, we used an optional iterative step by running a pilot DCE used to optimize the final DCE

Figure 1 provides a schematic overview of the study design, outlining the sequential steps from defining the clinical decision problem through vignette construction, the web‑based discrete‑choice experiment, statistical modelling, and integration into the BAIT system. From May 2022 till August 2023 during several sessions a representative number of intensivists of both hospitals (PT, JdM, BtT, RG, H-MF, BvdB) in collaboration with discrete choice experts (AtB, NH) defined criteria that intensivists would most likely use to decide whether they would (unconditionally) continue, continue during a time-limited trial or withdraw LST for patients admitted to the ICU. These criteria were subsequently operationalised as attributes in the discrete-choice experiment.

A web-based choice-experiment was designed, and participants were enrolled in January 2023. The choice-experiment scenarios were constructed using ChoiceDesign version 0.1.2 [17]. ChoiceDesign was used to generate an efficient experimental design based on the predefined attributes and levels, ensuring balanced representation and minimal correlation between attributes to ensure that choices made in the experiment would be as informative as possible concerning the weights assigned by intensivists. The resulting choice tasks were exported and presented to participants through a custom web-based questionnaire.

Feedback was used to improve the experiment by clarifying and refining the criteria and removing medically implausible combinations. An improved experiment was developed, and participants were enrolled from December 2023 to April 2024. The final design included 25 choice tasks per participant, consistent with standard practice in discrete‑choice experiments involving multiple attributes and levels [18]. All eligible intensivists and fellows at the participating centres were invited to participate, and the final sample reflects those who enrolled during the study period.

Representative example vignettes and the corresponding attribute-level tables are provided in the Supplementary Digital Content (Vignettes section).

Participants were characterised by role (intensivist or fellow) and by hospital (AUMC or OLVG). No additional demographic or cultural information was collected, as Dutch and EU privacy regulations restrict the processing of special categories of personal data unless strictly necessary for the research aim.

### Statistical analyses

Statistical analyses were performed using R Statistical Software v4.4.1 [19]. Multinomial and binary logistic regression models were developed using the Apollo package version 0.2.8 [20, 21]. In addition to an aggregate model using data from all participants, separate models were created for both participating hospitals (AUMC and OLVG), and for intensivists and fellows in intensive care medicine. For the binary model, the continue and time-limited continuation decision were combined. All models were specified as fixed‑coefficient logit models; no random parameters were included. Ordered categorical predictors were coded as integer scores (0, 1, 2, …) and entered as linear terms. To evaluate the linearity assumption, we estimated supplementary models with level‑specific coefficients for each ordered predictor (i.e., treating each ordered level as a separate category). Group differences were analysed using pooled interaction models, with a joint Wald test across all interaction terms as the primary inferential assessment, and Holm-adjusted individual Wald tests provided as complementary estimates of specific parameter differences. Relative factor importance was calculated using maximum utility contribution.

### Behavioural artificial intelligence technology system

The multinomial logistic regression model was used to develop a behavioural artificial intelligence system. By documenting the required criteria levels, intensivists were able to see the predicted decision as a percentage of their peers that would take that decision. In addition, color-coding was used to help visualize the direction (i.e. continue, time-limited-trial or withdraw) and magnitude of the effect for each documented criterion.

## Results

In the initial pilot experiment 23 intensivists (in training) completed the experiment. Plenary sessions were held discussing the pilot model and feedback was used to develop the final set of criteria (Table 1). Additionally, based on physician feedback, the narrative-based vignettes were replaced by forms explicitly showing the criteria reflecting the clinical status, prognostic factors and patient and family values of the fictitious patients to be evaluated (See Supplementary Digital Content, Vignettes section).

**Table 1:** Decision criteria and levels

	Level 0	Level 1	Level 2	Level 3
Baseline clinical and prognostic factors				
Expected additional ICU length of stay	A couple of weeks	A couple of months		
Clinical situation	Deterioration	No improvement		
Age (years)	40	55	70	85
Frailty at hospital admission	1–2	3–4	5–6	
Life expectancy (pre-admission)	6–12 months	1–5 years	> 5 years	
Burden of Suffering	Limited	Severe		
Expected post-ICU impairment				
Cardiovascular impairment	NYHA I	NYHA II	NYHA III	NYHA IV
Pulmonary impairment	No impairment	Moderate impairment	Severe impairment	
Renal impairment	No impairment (GFR > 30)	Pre-dialysis (GFR 15–30)	Dialysis dependent (GFR < 15)	
Neurological impairment	mRS 0–1	mRS 2–3	mRS 4–5	
Gastro-intestinal impairment	Without tube-feeding	Tube-feeding dependent		
Patient or family values				
Patient or family values	Expected future physical disabilities are possibly acceptable	Expected future physical disabilities are most likely unacceptable		

*NYHA* New York Heart Association classification of heart failure [31]. *GFR* Glomerular Filtration Rate. *mRS* Modified Rankin Scale [32, 33]. Definitions of the NYHA and mRS scales are available in Supplemental Digital Content, Tables S1 and S2

From December 2023 to April 2024 potential participants (n = 75) were invited by both oral invitation and e-mail. Thirty-six (48%) participants, 26 intensivists and 10 fellows in intensive care medicine fully completed the experiment containing 25 cases.

**Fig. 2:**
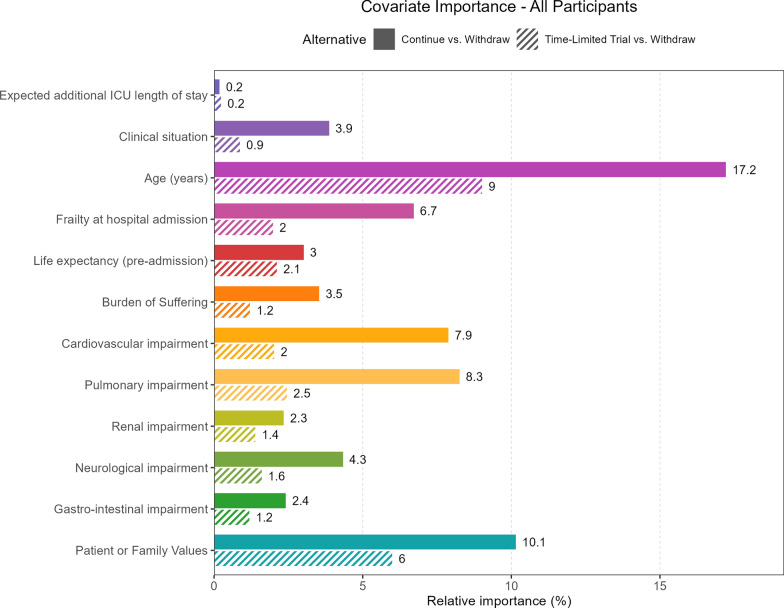
Relative importance of covariates for the multinomial model. Expected additional ICU length of stay did not appear to significantly influence the decision to continue or withdraw life-sustaining therapy

The estimated model consisted of twelve covariates and achieved good fit (McFadden’s *ρ*^2^ 0,25) [22]. Models with level-specific coefficients revealed that some predictors showed non-linearities across levels (Figure S1 and Table S3, Supplemental Digital Content). For consistency and interpretability, the final model retained the linear coding of ordered predictors. Multinomial coefficient weights for all participants are shown in Table 2. Relative importance of covariates is depicted in Fig. 2 and Supplementary Digital Content, Figure S2 for the multinomial and binary model, respectively. Age, patient and family values, expected cardiovascular and pulmonary impairment after ICU discharge and frailty at ICU admission were the most important covariates. Interestingly, expected ICU length of stay did not appear to significantly influence the decision of the consulted intensivists. Covariates associated with poorer prognosis—such as higher age, frailty, organ impairment, and greater burden of suffering—reduced the likelihood of choosing continuation and, to a lesser extent, reduced the likelihood of selecting a time-limited trial compared with withdrawal. Favourable indicators increased the likelihood of continuation more strongly than they increased the likelihood of selecting a time-limited trial.

**Table 2:** Coefficient weights and goodness of fit for the *all participants* and subgroup models

Coefficient weights across all groups											
	Alternative	All participants		Amsterdam UMC		OLVG		Intensivists		Fellows	
		Weight [95% CI]	*p* value	Weight [95% CI]	*p* value	Weight [95% CI]	*p* value	Weight [95% CI]	*p* value	Weight [95% CI]	*p* value
Criteria											
Expected additional ICU length of stay	Continue^*1*^	− 0.0589 [− 0.565, 0.448]	0.82	− 0.316 [− 1.017, 0.385]	0.377	0.303 [− 0.415, 1.022]	0.408	− 0.101 [− 0.701, 0.498]	0.74	− 0.228 [− 1.300, 0.844]	0.677
	Time-Limited^*2*^	− 0.0757 [− 0.356, 0.205]	0.597	− 0.185 [− 0.556, 0.187]	0.33	0.0324 [− 0.390, 0.454]	0.881	− 0.222 [− 0.592, 0.148]	0.239	0.208 [− 0.127, 0.543]	0.224
Clinical situation	Continue^*1*^	**1.25 [0.589, 1.911]**	0.000211	**1.06 [0.316, 1.811]**	0.00528	**1.49 [0.201, 2.785]**	0.0235	**0.824 [0.090, 1.559]**	0.0278	**2.92 [1.642, 4.199]**	< 0.0001
	Time-Limited^*2*^	0.282 [− 0.298, 0.862]	0.341	0.133 [− 0.606, 0.871]	0.724	0.491 [− 0.493, 1.475]	0.328	0.0228 [− 0.612, 0.658]	0.944	1.41 [− 0.164, 2.985]	0.079
Age (years)	Continue^*1*^	− **1.85 [**− **2.278, **− **1.429]**	< 0.0001	− **1.73 [**− **2.187, **− **1.278]**	< 0.0001	− **2.1 [**− **2.947, **− **1.262]**	< 0.0001	− **1.76 [**− **2.224, **− **1.300]**	< 0.0001	− **2.79 [**− **3.567, **− **2.006]**	< 0.0001
	Time-Limited^*2*^	− **0.971 [**− **1.221, **− **0.721]**	< 0.0001	− **0.922 [**− **1.251, **− **0.594]**	< 0.0001	− **1.04 [**− **1.448, **− **0.625]**	< 0.0001	− **0.956 [**− **1.253, **− **0.658]**	< 0.0001	− **1.18 [**− **1.673, **− **0.677]**	< 0.0001
Frailty at hospital admission	Continue^*1*^	− **1.08** **[**− **1.609, **− **0.560]**	< 0.0001	− **1.04 [**− **1.610, **− **0.463]**	0.000393	− **1.24 [**− **2.338, **− **0.147]**	0.0263	− **0.699 [**− **1.235, **− **0.163]**	0.0106	− **3.08 [**− **3.953, **− **2.208]**	< 0.0001
	Time-Limited^*2*^	− 0.32 [− 0.651, 0.011]	0.0578	− 0.332 [− 0.767, 0.103]	0.134	− 0.326 [− 0.826, 0.173]	0.2	− 0.0906 [− 0.445, 0.264]	0.616	− **1.25[**− **2.046, **− **0.448]**	0.00222
Life expectancy (pre-admission)	Continue^*1*^	**0.487 [0.181, 0.792]**	0.00179	**0.418 [0.017, 0.820]**	0.0411	**0.564 [0.048, 1.079]**	0.0321	**0.638 [0.237, 1.039]**	0.00182	0.0493 [− 0.637, 0.736]	0.888
	Time-Limited^*2*^	**0.341 [0.108, 0.574]**	0.00407	**0.355 [0.048, 0.662]**	0.0234	0.351 [− 0.030, 0.732]	0.0712	**0.409 [0.102, 0.716]**	0.00909	0.252 [− 0.150, 0.654]	0.22
Burden of Suffering	Continue^*1*^	− **1.14 [**− **1.979, **− **0.304]**	0.00753	− **1.19 [**− **2.064, -0.309]**	0.00805	− 1.13 [− 2.673, 0.411]	0.151	− 0.716 [− 1.621, 0.188]	0.121	**-3.26 [**− **5.302, **− **1.220]**	0.00174
	Time-Limited^*2*^	− 0.391 [− 1.048, 0.266]	0.243	− 0.659 [− 1.462, 0.144]	0.108	− 0.102 [− 1.205, 1.001]	0.856	− 0.0599 [− 0.791, 0.671]	0.872	− **1.63 [**− **2.872, **− **0.379]**	0.0106
Cardiovascular impairment	Continue^*1*^	− **0.849 [**− ** 1.119, **− **0.580]**	< 0.0001	− **0.795 [**− **1.125, **− **0.465]**	< 0.0001	− **0.95 [**− **1.507, **− **0.393]**	0.000834	− **0.771 [**− **1.063, **− **0.480]**	< 0.0001	− **1.6 [**− **2.372, **− **0.833]**	< 0.0001
	Time-Limited^*2*^	− **0.218[**− **0.372, **− **0.065]**	0.00532	− 0.19 [− 0.445, 0.065]	0.144	− **0.25 [**− **0.426, **− **0.074]**	0.00545	− **0.263 [**− **0.445, **− **0.082]**	0.00448	− 0.22 [− 0.557, 0.117]	0.2
Pulmonary impairment	Continue^*1*^	− **1.33 [**− **1.839, **− **0.828]**	< 0.0001	− **1.49 [**− **2.109, **− **0.872]**	< 0.0001	− **1.14** **[**− **2.068, -0.203]**	0.017	− **1.07 [**− **1.639, **− **0.494]**	0.000262	− **2.71 [**−**3.458, -1.953]**	< 0.0001
	Time-Limited^*2*^	− **0.396** **[**− **0.693, **− **0.100]**	0.00877	− **0.479 [**− **0.865, **− **0.094]**	0.0149	− 0.298[− 0.783, 0.187]	0.228	− 0.291 [− 0.653, 0.070]	0.114	− **0.832 [**−**1.346, **−**0.318]**	0.00152
Renal impairment	Continue^*1*^	− 0.379 [− 0.853, 0.095]	0.117	− **0.597 [-1.097, **− **0.098]**	0.0191	− 0.0184 [− 0.873, 0.836]	0.966	− 0.312 [− 0.901, 0.277]	0.299	− 0.344 [−0.814, 0.126]	0.152
	Time-Limited^*2*^	− 0.224 [− 0.519, 0.071]	0.137	− 0.173 [− 0.462, 0.115]	0.239	− 0.283 [− 0.829, 0.263]	0.31	− 0.223 [− 0.577, 0.132]	0.219	−0.417[− 0.943, 0.109]	0.121
Neurological impairment	Continue^*1*^	− **0.7 [**− **1.267, -0.134]**	0.0153	− **0.754 [**− **1.395, **− **0.113]**	0.0212	− 0.73 [− 1.689, 0.230]	0.136	− 0.37 [− 1.000, 0.261]	0.251	− **2.29 [**− **3.533, **− **1.044]**	0.000313
	Time-Limited^*2*^	− 0.259 [− 0.725, 0.208]	0.277	− 0.301 [− 0.843, 0.241]	0.276	− 0.229 [− 1.017, 0.560]	0.57	− 0.0461 [− 0.562, 0.470]	0.861	− 1.18 [− 2.366, 0.002]	0.0504
Gastro-intestinal impairment	Continue^*1*^	− **0.778 [**− **1.296, **− **0.261]**	0.00319	− **0.945 [**− **1.621, -0.270]**	0.00608	−0.557 [−1.315, 0.201]	0.15	− **0.773 [**− **1.397,**− **0.149]**	0.0151	− 0.787 [− 1.598, 0.024]	0.0571
	Time-Limited^*2*^	− 0.383 [− 0.775, 0.009]	0.0556	− 0.474 [− 0.992, 0.044]	0.0728	− 0.303 [− 0.895, 0.288]	0.315	− 0.349 [− 0.782, 0.083]	0.113	− 0.763 [− 1.891, 0.365]	0.185
Patient or Family values	Continue^*1*^	− **3.28 [**− **4.171, -2.385]**	< 0.0001	− **3.21 [**− **4.277, **− **2.137]**	< 0.0001	− **3.52 [**− **5.079, **− **1.964]**	< 0.0001	− **2.88 [-3.896, -1.856]**	< 0.0001	− **5.06 [**− **6.810, **− **3.319]**	< 0.0001
	Time-Limited^*2*^	− **1.94 [**− **2.614, -1.259]**	< 0.0001	− **1.88 [**− **2.678, **− **1.075]**	< 0.0001	− **2.07 [**− **3.261, **− **0.878]**	0.000662	− **1.51 [**− **2.188, **− **0.826]**	< 0.0001	− **3.78 [**− **5.734, **− **1.836]**	0.000141
Constant	Continue^*1*^	**7.28 [4.657, 9.907]**	< 0.0001	**7.84 [5.125, 10.557]**	< 0.0001	**6.9 [1.737, 12.055]**	0.0088	**5.74 [2.906, 8.581]**	< 0.0001	**15.7 [10.524, 20.822]**	< 0.0001
	Time-Limited^*2*^	**3.55 [1.617, 5.477]**	0.000315	**3.78 [1.562, 6.000]**	0.000841	**3.35 [0.065, 6.633]**	0.0456	**2.7 [0.544, 4.854]**	0.0141	**7.66 [3.630, 11.696]**	0.000196
Goodness of fit											
Null log-likelihood			− 989		− 522		− 467		− 714		− 275
Log-likelihood of estimated model			− 716		− 384		− 324		− 519		− 180
Adjusted${\rho }^{2}$			0.25		0.215		0.25		0.236		0.248

^*1*^Continue: Continue life-sustaining therapy vs. Withdrawal

^*2*^Time-Limited: Time-Limited Trial vs. Withdrawal

Bold = *p* < 0.05. Coefficients: Positive = Effect towards Continue/Time-Limited Trial, Negative = Effect towards Withdraw,

### Subgroup analysis

**Fig. 3:**
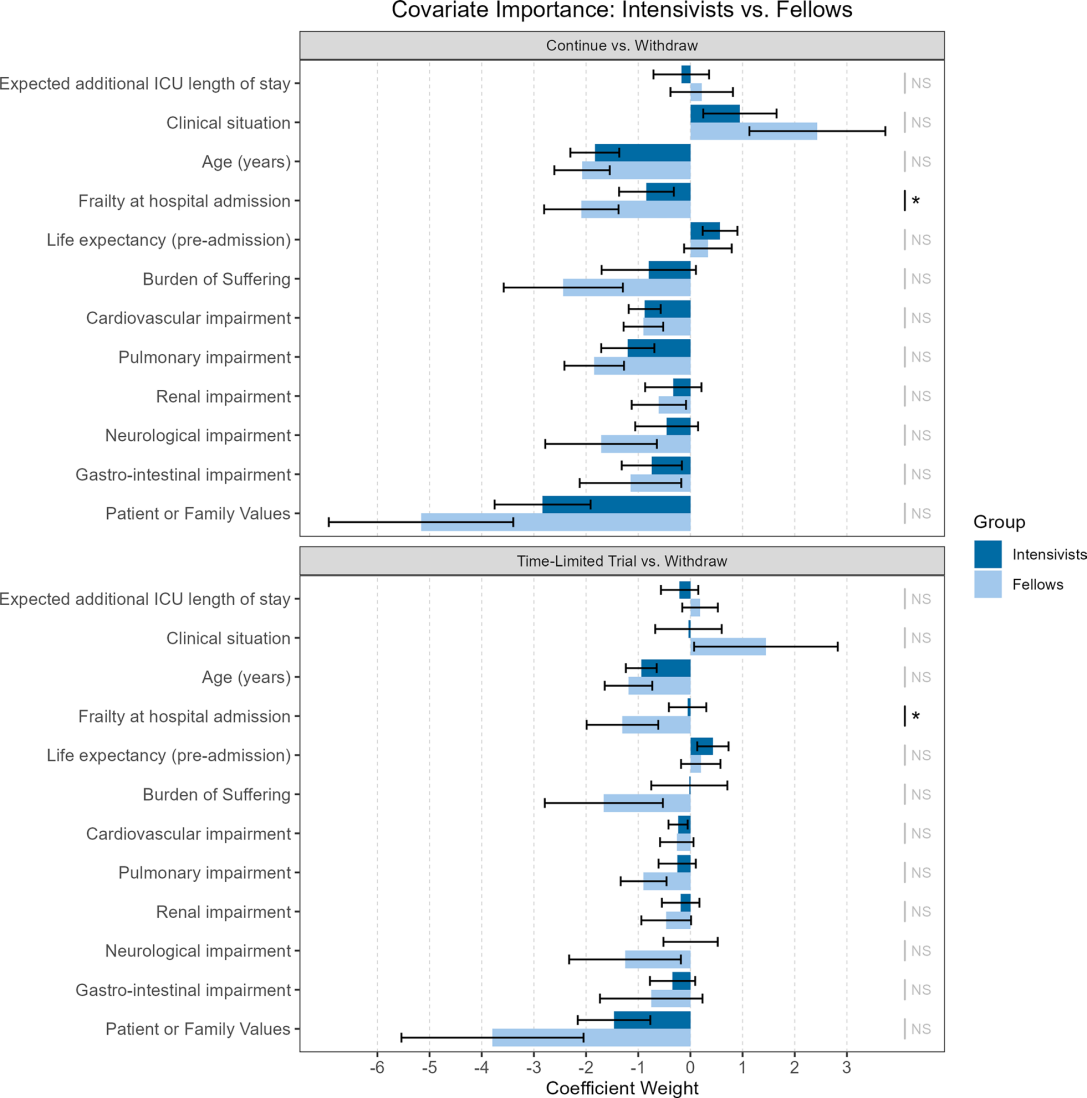
Comparison of covariate importance. Covariate importance is expressed as coefficient weights. Differences in coefficient weights were tested using the Wald test in a pooled interaction model of both groups. Error bars show 95% confidence intervals. *NS* not statistically significant. *p < 0.05

When comparing the preferences of participants of both hospitals using a pooled model, no statistically significant differences were observed (Table 3 and Supplementary Digital Content, Figure S3). However, overall, the preferences of intensivists versus fellows showed a statistically significant difference, with a higher impact of frailty at hospital admission for the fellow group (Table 3 and Fig. 3). Other covariates did not demonstrate statistically significant differences between the groups.

**Table 3:** Subgroup comparison—multinomial pooled interaction models

	Amsterdam UMC vs. OLVG			Intensivists vs. Fellows		
Criteria	Wald statistic	Uncorrected *p* value	*p* value	Wald statistic	Uncorrected *p* value	*p* value
Overall group difference	15.1		0.368	50.1***		< 0.0001
Individual criteria						
Expected additional ICU length of stay	1.67	0.196	1	2.47	0.116	0.695
Clinical situation	0.512	0.474	1	3.92	0.0477	0.334
Age (years)	0.276	0.6	1	0.797	0.372	1
Frailty at hospital admission	0.00193	0.965	1	10.8*	0.00101	0.0141
Life expectancy (pre-admission)	0.0141	0.906	1	0.946	0.331	1
Burden of Suffering	0.374	0.541	1	5.96	0.0147	0.152
Cardiovascular impairment	0.0874	0.767	1	0.0175	0.895	1
Pulmonary impairment	0.662	0.416	1	5.53	0.0187	0.168
Renal impairment	0.000173	0.99	1	0.842	0.359	1
Neurological impairment	0.00706	0.933	1	4.47	0.0344	0.275
Gastro-intestinal impairment	0.311	0.577	1	0.59	0.443	1
Patient or Family values	0.127	0.722	1	6.06	0.0138	0.152
Constant—Continue^*1*^	0.201	0.654	1	6.29	0.0122	0.146
Constant—Time-Limited^*2*^	0.0654	0.798	1	6.49	0.0109	0.141

^*^ *p* < 0.05; *** *p* < 0.001

^1^Continue: Continue life-sustaining therapy vs. Withdrawal

^2^Time-Limited: Time-Limited Trial vs. Withdrawal

### Behavioural artificial intelligence technology system

**Fig. 4:**
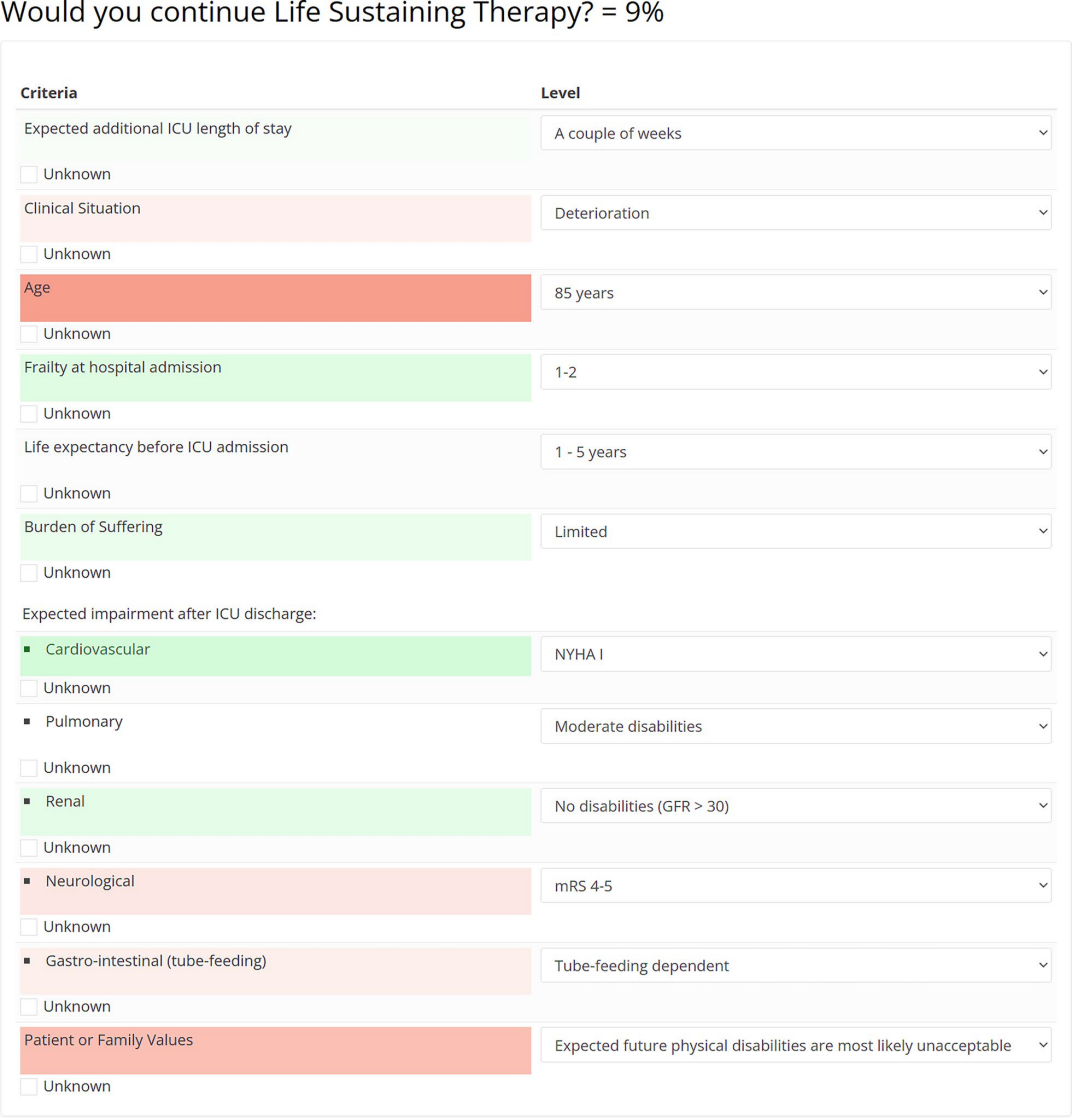
Web-based behavioural artificial intelligence system. After filling out the required criteria levels the system calculates the number of intensivists that would have made the same decision. In this example only 9% of intensivists would have decided to continue life-sustaining therapy. Colour-coding is used to intuitively show the direction, with green for continue and red for discontinue, and the magnitude of the effect by the intensity of the colour for that criterion

The resulting BAIT system translated the estimated model into an interactive interface that displays predicted decisions and visualises attribute effects (Fig. 4).

## Discussion

In this study we developed a discrete choice model that helps to elucidate the criteria influencing the decision to continue or withdraw LST of intensivists and fellows in intensive care medicine of two Dutch urban hospitals.

Age has traditionally been used a marker for risk assessment. However, the response to advancing age varies and recent advancements lead to the development of the concept of frailty syndrome [23]. With the inclusion of frailty at hospital admission in our model, we expected a relatively large effect of frailty, but age still appeared to be more significant on the decision to continue LST (Fig. 2). A possible explanation might be that while frailty influences the decision to admit patients to the ICU, age is used to determine proportionality in continuing treatment for already admitted patients.

Patient values (often represented by family or relatives) played an important role in continuing or withdrawing in our experiment, reflecting the importance in ICUs of family centred care with shared decision-making. The relatively small effect of neurological impairment may have been offset by the large role of patient values, suggesting that intensivists require a proxy of the patient to determine whether a neurological outcome is acceptable.

Interestingly, while in sessions with intensivists it was suggested that the expected length of stay would influence the decision, since it was assumed this was a proxy of the severity of the underlying illness, in the experiment this did not appear to influence the chosen alternative. Within our discrete-choice design, expected length of stay was included following recommendations from participating physicians. Although its interpretability may be less clear in scenarios lacking explicit diagnostic or severity details, clinicians noted that expected length of stay is commonly used to gauge clinical trajectory and resource allocation. We therefore retained the covariate to reflect clinical judgement and preserve consistency in the experimental design. Nevertheless, prior studies have shown that clinicians’ ability to predict clinical trajectories, including the need for prolonged mechanical ventilation, is limited [24]. Intensivists may recognise the limited reliability of such predictions and consequently place less weight on expected additional ICU length compared with more robust indicators such as age, frailty, organ dysfunction, and patient values. Within the vignette structure, expected length of stay may also lack the contextual cues clinicians typically use to interpret prognostic estimates, further reducing its perceived relevance.

The multinomial model also clarifies the circumstances under which clinicians preferred a time-limited trial. The graded pattern across attributes suggests that time-limited trials were most often selected in situations of intermediate or unresolved prognostic uncertainty, where full continuation appeared disproportionate but immediate withdrawal felt premature. In this sense, the model reflects how time-limited trials function in clinical practice as a structured approach to managing uncertainty, allowing clinicians to reassess the patient’s trajectory before committing to longer-term treatment decisions.

While there were no significant differences in the models of the two hospitals, there was a significant difference between the coefficient weights of intensivists vs. fellows (Table 3). However, only frailty had a statistically significant higher effect on the decisions of fellows, though as mentioned, for all groups, age still appears the most important covariate.

Compared to conventional rule-based or knowledge-based approaches, the discrete choice approach allows choices during the experiment to reveal expertise. Especially for difficult ethical decisions, individuals may find it challenging to describe why they made a certain decision, partly because these decisions are made by intuition or unconsciously [25, 26]. By modelling their decisions, experts are not required to explicitly state their rationale, and arbitrary deterministic rules are not needed.

While machine learning based models trained on large historic datasets may offer a seemingly attractive alternative, many of the best performing algorithms are difficult to understand. Since explainable models are important for achieving confidence by providers, it could be argued that models are ideally fully interpretable [27]. The coefficient weights used in BAIT are relatively easy to interpret and allow a clear understanding of the factors leading to a decision. In addition, the currently available historic ICU datasets do not contain the factors intensivists use to make their decision impeding model development and validation [28].

Our study also has several limitations. Firstly, since our results were generated in a single academic medical centre and one large urban teaching hospital in the Netherlands, they may not generalize well to smaller or non-teaching hospitals and especially health organisations outside of the Netherlands. The generalizability of our findings is also shaped by the Dutch legal and cultural context in which decisions about withholding or withdrawing LST are made. Concepts such as medical futility, the role of patient representatives, and the structure of shared decision-making vary substantially across jurisdictions and religious traditions. The BAIT framework can be adapted to other settings by modifying attribute definitions, incorporating locally relevant ethical or legal considerations, and involving clinicians and patient representatives from the target context during vignette development. In addition, the choice alternatives themselves may require adaptation. Whereas the Dutch context allows decisions between continuing treatment, continuing with a time-limited trial, or withdrawing treatment, other jurisdictions or religious traditions may restrict the ethically or legally permissible options. In such settings, the framework could instead focus on choices such as withholding treatment escalation versus continuing or escalating treatment. These adaptations would allow the discrete-choice experiment to reflect the normative, cultural, and legal environment of other countries or religious communities while maintaining the methodological structure of the approach. A further limitation is that we did not collect demographic or cultural characteristics of respondents beyond their role (intensivist or fellow) and participating hospital. Under EU and Dutch privacy legislation—specifically the General Data Protection Regulation (GDPR, Article 9) and its national implementation in the Uitvoeringswet AVG (UAVG)—data on ethnicity, cultural background, and religion are classified as special categories of personal data and may only be processed when strictly necessary for the research aim. As these characteristics were not essential to the study objectives, they were not collected. Although all participants worked within the same national healthcare and legal framework, unmeasured heterogeneity in personal background, clinical experience, or values may still have influenced individual decision-making.

Secondly, although we aimed to quantify the effect several criteria have on the decision-making process and used several sessions to compile the list of potential factors, it cannot be ruled out that other criteria may play a role in establishing futility of treatment and thus influence the decision to continue or withdraw LST. In real-world practice, decisions about LST are also shaped by contextual influences that were not included in our experiment, such as culture, local policy, staffing levels, bed strain, prior family meetings, religious beliefs, litigation experience, and diagnosis-specific clinical trajectories. These criteria can meaningfully affect how intensivists interpret clinical information and engage in shared decision-making. Because such influences are highly variable across settings and difficult to standardize within a discrete-choice experiment, they were not incorporated into the model. We therefore emphasise that BAIT should not be viewed as a replacement for clinical judgement or the broader contextual information available at the bedside. Instead, the tool is intended to make explicit the relative importance of the clinical and value-based criteria included in the experiment, while recognising that clinicians must continue to integrate organisational, cultural, and situational factors when applying the model in practice. A further consideration is that decisions about LST are inherently dynamic. In clinical practice, intensivists repeatedly reassess the patient’s trajectory, response to treatment, evolving organ dysfunction, and the outcomes of prior family meetings. These iterative evaluations often carry as much weight as the initial clinical review. Our discrete-choice experiment, by design, captures a single moment of decision-making and therefore cannot fully reflect the temporal dimension through which prognostic uncertainty resolves and treatment goals are revisited. This limitation is intrinsic to vignette-based approaches and should be kept in mind when interpreting the model outputs, as real-world decisions are shaped by evolving clinical information rather than static presentations.

A further methodological limitation is the assumption of linearity in several ordered covariates. Supplementary models showed non‑linear patterns for predictors such as age, life expectancy, pulmonary impairment, and renal impairment. For simplicity and interpretability, we retained a linear specification in the final multinomial model. The results should therefore be interpreted with the understanding that some covariates may exert non‑linear influences that are not fully captured.

Additionally, while BAIT allowed us to help elucidate the importance of criteria influencing our decision, it cannot answer the question why those criteria were considered relevant. Since coefficient weights reflect decisions of a group of physicians, BAIT may unintentionally reinforce majority opinions. Large studies evaluating peer pressure on physician end-of-life decisions are lacking, but a network study does suggest at least a modest effect [29]. In part this is mitigated in the system by showing the percentage of intensivists choosing a specific strategy and not giving the strategy directly as an advice to follow. Nevertheless, physicians need to carefully consider how to use BAIT in clinical practice while still taking minority voices into account.

Many medical societies and associated law dictate that the professional standard determines whether treatment is considered medically futile [5, 8]. But as part of shared decision-making patient beliefs should be integral part of these discussions. In similar discrete choice experiments with the general population, physicians and nurses, the general population was more likely to choose to withdraw LST [16, 30]. We therefore recommend validating our model in the general population and with ICU patient associations and support communities to identify discrepancies. Discrepancies are not inherently problematic; however, they underscore the importance of maintaining an open approach during meetings with representatives of critically ill patients.

Finally, the cases were hypothetical. Despite substantial efforts to make the cases representative of clinical practice, a significant limitation in their evaluation was the absence of a specific condition or disease, which would hinder any generalisability beyond that particular illness.

## Conclusions

We developed a clinical decision support system using behavioural artificial intelligence technology for the dilemma to continue or withdraw life-sustaining therapy. Elucidating and quantifying the criteria involved in these decisions may clarify the inherently subjective nature of medical futility.

## Take-home message

Behavioural Artificial intelligence Technology is a novel technique that helps elucidate the key criteria involved in end-of-life decisions. A BAIT system may support decisions to continue or withdraw life-sustaining therapies in the ICU.

## Supplementary Information

Supplementary Material 1.

## Data Availability

Respondent data, full code of the analyses, and the developed binary and multinomial models are available at https://github.com/patrickthoral/baitlist.

## Abbreviations

AI: Artificial Intelligence
AUMC: Amsterdam University Medical Center
BAIT: Behaviour artificial intelligence technology
GFR: Glomerular filtration rate
ICU: Intensive care unit
LST: Life-sustaining therapies
NS: Not statistically significant
NYHA: New York Heart Association (classification of heart failure)
mRS: Modified rankin score
SE: Standard error
