# Supplementary material for: Behaviour artificial intelligence technology to support the decision-making process of continuation or withdrawal of life-sustaining therapy
Source: Intensive Care Med Exp. 2026 Jul 1;14:84. doi: 10.1186/s40635-026-00930-4 (PMC13323472; doi:10.1186/s40635-026-00930-4)
Supplement: Supplementary file 1 — Supplementary Material 1. [file 40635_2026_930_MOESM1_ESM.pdf]

# Supplementary Digital Content

## Behaviour Artificial Intelligence Technology to support the decision-making process of continuation or withdrawal of life sustaining therapy

Patrick J. Thoral, Jesse de Metz, Stella Mulia, Annebel ten Broeke, Nicolaas Heyning, Birkitt L. ten Tusscher, Rolf K. Gigengack, Hilde M. Feijen, Caspar G. Chorus, Bas van den Bogaard, Paul W.G. Elbers

### Contents

Vignettes ..... 2

Pilot Study vignette ..... 2

English Translation ..... 2

English Translation of Table of criteria levels used in pilot vignette ..... 3

Original Dutch vignette text ..... 4

Original Dutch Table of criteria levels used in pilot vignette ..... 5

Study Vignette ..... 6

English Translation of vignette text ..... 6

Original Dutch vignette text ..... 7

Table S1. New York Heart Association classification of heart failure ..... 8

Table S2. The Modified Rankin Scale ..... 9

Figure S1. Ordered covariates using level-specific coefficients ..... 10

Table S3. Linearity Diagnostics for Ordered Covariates ..... 11

Figure S2. Relative importance of covariates for the binary model ..... 12

Figure S3. Comparison of Covariate Importance ..... 13

# Vignettes

## Pilot Study vignette

### English Translation

Patient X has been treated in your ICU for {a}, during which cardiopulmonary support has {m}. The treatment team has begun to question whether it remains meaningful to continue treatment aimed at recovery.

The patient is {b} years old, had an estimated frailty score of {c} at admission, and was admitted with a condition for which the expected life expectancy of the underlying disease was {d}.

At this point, there is {e} burden of suffering. The estimation is that it will take {f} before the patient will function approximately at the pre-existing level again.

It is expected that the following impairments will be present at that time: heart failure, {g} lung failure, leading to {h} kidney failure, leading to {i} and the patient is expected to function cognitively at {j}. There will be {k} dependence on tube feeding or TPN.

During previous admissions, the expected burden of suffering and future impairments were discussed repeatedly. In conversations with the family, it became clear that (l) for the patient and their loved ones.

Would you continue treatment aimed at recovery? Choice:

- Yes, full treatment
- Yes, but without escalation
- No, withdraw treatment

## English Translation of Table of criteria levels used in pilot vignette

| Criteria | Level 1 | Level 2 | Level 3 | Level 4 |
|---|---|---|---|---|
| **a** | A few days | A few weeks | A few months | |
| **m** | increases | remains stable | decreases | |
| **b** | 40 | 55 | 70 | 85 |
| **c** | 1-2 | 3-4 | 5-6 | 7 |
| **d** | years | months to a year | weeks to months | |
| **e** | limited | moderate to severe | very severe | |
| **f** | less than 3 months | >3 months to a year | | |
| **g** | NYHA I | NYHA II | NYHA III | NYHA IV |
| **h** | No limitations | Moderate limitations | Severe limitations | |
| **i** | No limitations (GFR > 30) | pre-dialysis phase (GFR 15-30) | Dialysis (GFR <15) | |
| **j** | mRS (modified Rankin Scale) 0-1 | mRS 2-3 | mRS 4-5 | |
| **k** | without | with | | |
| **l** | the expected future limitations are acceptable | certain expected future limitations are acceptable | the expected future limitations are unacceptable | |

## Original Dutch vignette text

Patiënt X wordt al {a} behandeld op uw IC, waarbij de cardiopulmonale ondersteuning {m}. Het behandelteam is gaan twijfelen of het zinvol is om behandeling gericht op herstel voort te zetten.

Patiënt is {b} jaar oud, had bij opname een ingeschatte frailty van {c} en werd opgenomen met een aandoening waarvan de verwachte levensduur van het onderliggende ziektebeeld {d} zou zijn.

Inmiddels is er sprake van {e} lijdenslast. De inschatting is dat {f} zal duren voordat patiënt weer ongeveer op het pre-existente niveau zal functioneren.

De verwachting is dat er dan sprake zal zijn van de volgende beperkingen:

hartfalen, {g}

longfalen, leidend tot {h}

nierfalen, leidend tot {i}

waarbij patiënt cognitief zal functioneren op {j}

{k} afhankelijkheid van sondevoeding of TPV

Bij eerdere opnames is herhaaldelijk gesproken over de verwachte lijdenslast en toekomstige beperkingen.

In gesprekken met familie werd duidelijk dat (l) voor patient en zijn naasten.

Zou u de behandeling gericht op herstel voortzetten?

- Ja volledige behandeling
- Ja, zonder escaleren
- Nee, stoppen

## Original Dutch Table of criteria levels used in pilot vignette

| Criteria | Level 1 | Level 2 | Level 3 | Level 4 |
|---|---|---|---|---|
| **a** | Enkele dagen | Enkele weken | Enkele maanden | |
| **m** | toeneemt | stabiel blijft | afneemt | |
| **b** | 40 | 55 | 70 | 85 |
| **c** | 1-2 | 3-4 | 5-6 | 7 |
| **d** | jaren | maanden tot een jaar | weken tot maanden | |
| **e** | beperkte | Matig tot ernstige | Zeer ernstige | |
| **f** | minder dan 3 maanden | >3 maanden tot een jaar | | |
| **g** | NYHA I | NYHA II | NYHA III | NYHA IV |
| **h** | Geen beperkingen | Matige beperkingen | Ernstige beperkingen | |
| **i** | Geen beperkingen (GFR > 30) | pre-dialyse traject (GFR 15-30) | Dialyse (GFR <15) | |
| **j** | mRS (modified rankin scale) 0-1 | mRs 2-3 | mRS 4-5 | |
| **k** | zonder | met | | |
| **l** | de verwachte toekomstige beperkingen acceptabel zijn | bepaalde verwachte toekomstige beperkingen acceptabel zijn | de verwachte toekomstige beperkingen onacceptabel zijn | |

## Study Vignette

In contrast to the pilot experiment, the study vignette showed criteria and the values (see Table 1) for the patient explicitly.

### English Translation of vignette text

Criteria:

1. Expected ICU admission duration
2. Clinical expectation
3. Age
4. Frailty at hospital admission
5. Life expectancy prior to ICU admission based on comorbidity
6. Current burden of suffering (as assessed by the treatment team)
7. It is anticipated that the following limitations will be present after ICU admission:
    a. Cardiac
    b. Pulmonary
    c. Renal
    d. Neurological
    e. Gastrointestinal (tube feeding)
8. Patient and/or family wishes

Would you continue treatment aimed at recovery?

- Yes
- Yes, with a Time-Limited Trial
- No, discontinue

## Original Dutch vignette text

Criteria:

1. Verwachte IC-opnameduur
2. Klinische verwachting
3. Leeftijd
4. Frailty bij ziekenhuisopname
5. Op basis van de comorbiditeit was de levensverwachting vóór IC opname
6. Actuele lijdenslast (inschatting door het behandelteam)
7. De verwachting is dat er na de IC-opname sprake zal zijn van de volgende beperkingen:
    a. Cardiaal
    b. Pulmonaal
    c. Renaal
    d. Neurologisch
    e. Gastro-intestinaal (sonde-voeding)
8. Wens patiënt en/of familie

Zou u de behandeling gericht op herstel voortzetten?

- Ja
- Ja, met Time Limited Trial
- Nee, stoppen

## Table S1. New York Heart Association classification of heart failure

| Class | Description |
|---|---|
| **I** | Patients with cardiac disease, but without resulting limitation of physical activity. Ordinary physical activity does not cause undue fatigue, palpitation, dyspnea, or anginal pain. |
| **II** | Patients with cardiac disease resulting in slight limitation of physical activity. They are comfortable at rest. Ordinary physical activity results in fatigue, palpitation, dyspnea, or anginal pain. |
| **III** | Patients with marked limitation of physical activity. They are comfortable at rest. Less than ordinary activity causes fatigue, palpitation, dyspnea, or anginal pain. |
| **IV** | Patients with cardiac disease resulting in inability to carry on any physical activity without discomfort. Symptoms of heart failure or of the anginal syndrome may be present even at rest. If any physical activity is undertaken, discomfort is increased. |

The New York Heart Association (NYHA) classification system categorizes the severity of heart failure into four classes, ranging from Class I (no limitation) to Class IV (severe symptoms at rest)

## Table S2. The Modified Rankin Scale

| Grade | Description |
|---|---|
| **0** | No symptoms at all |
| **1** | No significant disability despite symptoms: able to carry out all usual duties and activities |
| **2** | Slight disability: unable to carry out all previous activities but able to look after own affairs without assistance |
| **3** | Moderate disability: requiring some help, but able to walk without assistance |
| **4** | Moderately severe disability: unable to walk without assistance, and unable to attend to own bodily needs without assistance |
| **5** | Severe disability: bedridden, incontinent, and requiring constant nursing care and attention |

The Modified Rankin Scale is a six-point scale that categorizes the degree of neurological disability into six grades, ranging from Grade 0 (no disability) to Grade 5 (severe disability)

## Figure S1. Ordered covariates using level-specific coefficients

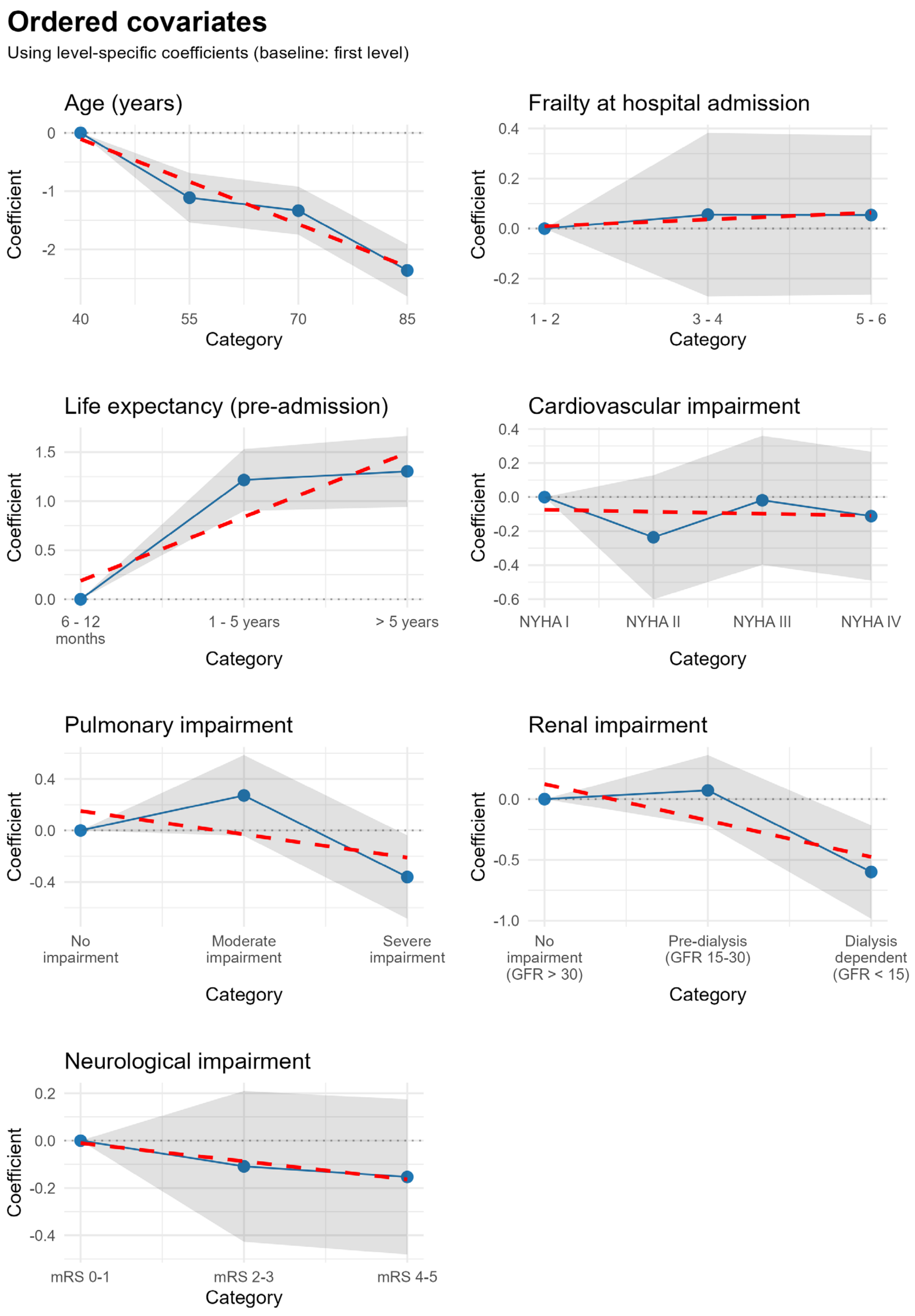


**Figure S1. Ordered covariates using level-specific coefficients.** Visualizes coefficients for each discretely encoded category.

## Table S3. Linearity Diagnostics for Ordered Covariates

Linearity Diagnostics for Ordered Covariates

| **Covariate** | **Likelihood-ratio test *(p)*[1]** | **Quadratic term *(p)*[2]** | **AIC: linear model[3]** | **AIC: categorical model[3]** | **Preferred functional form** |
|---|---|---|---|---|---|
| Age (years) | 0.020 | 0.934 | 1128.3 | 1124.5 | Non-linear |
| Frailty at hospital admission | 0.842 | 0.842 | 1249 | 1251 | Linear |
| Life expectancy (pre-admission) | < 0.001 | < 0.001 | 1187.6 | 1173.9 | Non-linear |
| Cardiovascular impairment | 0.360 | 0.548 | 1249.1 | 1251 | Linear |
| Pulmonary impairment | 0.002 | 0.002 | 1245.5 | 1237.8 | Non-linear |
| Renal impairment | 0.012 | 0.013 | 1243.4 | 1239.1 | Non-linear |
| Neurological impairment | 0.819 | 0.819 | 1248.3 | 1250.2 | Linear |

[1] Likelihood-ratio test comparing linear vs. level-specific coefficients model.

[2] Wald test for the quadratic ($x^2$) term in a polynomial model.

[3] Akaike Information Criterion; lower values indicate better relative model support.

**Table S3. Linearity Diagnostics for Ordered Covariates**. Demonstrates validity of linearity assumptions using the likelihood-ration test comparing linear vs. level-specific coefficients model, the Wald test for the quadratic ($x^2$) term in a polynomial model and the Akaike Information Criterion (AIC).

## Figure S2. Relative importance of covariates for the binary model

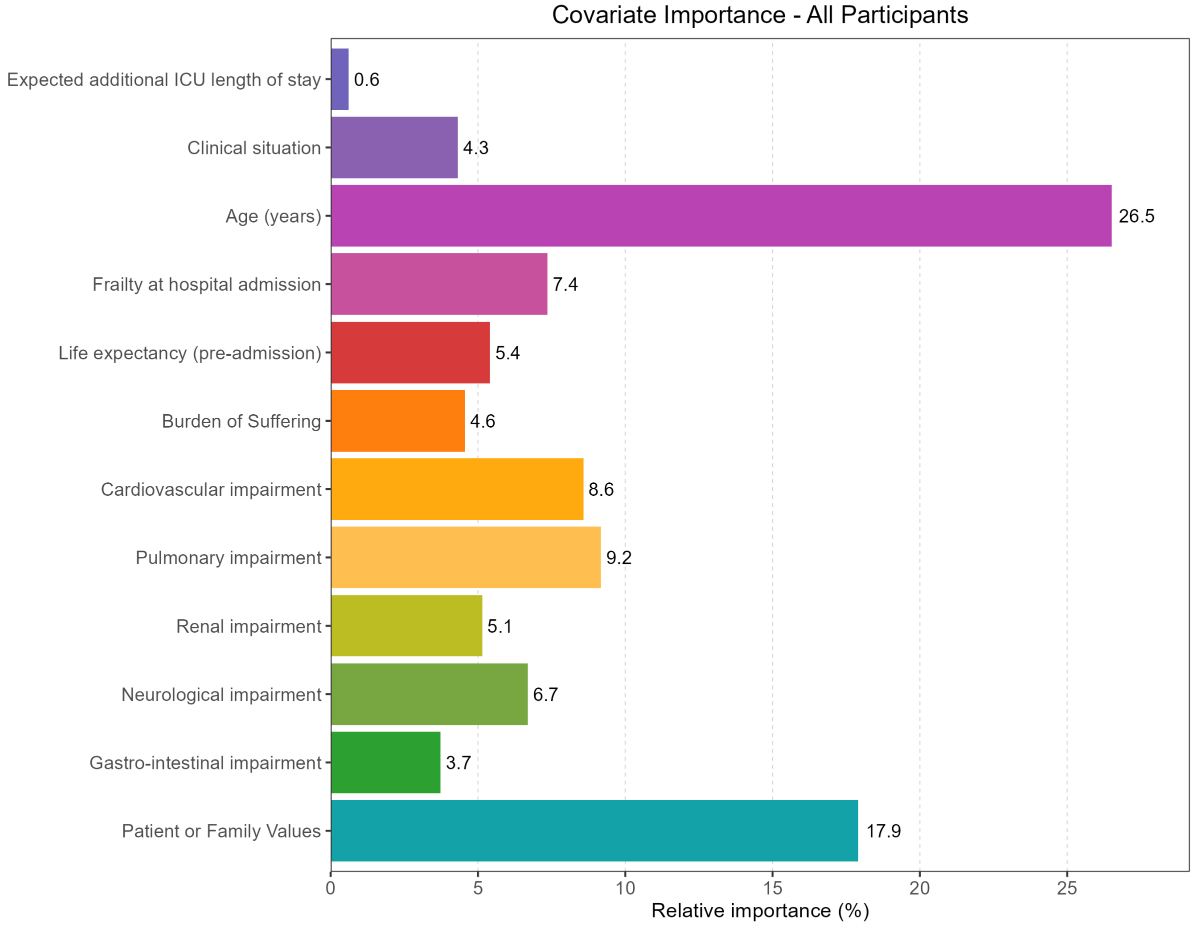


**Figure S2. Relative importance of covariates for the binary model**.
Expected additional ICU length of stay did not appear to significantly influence the decision to continue or withdraw life-sustaining therapy.

# Figure S3. Comparison of Covariate Importance

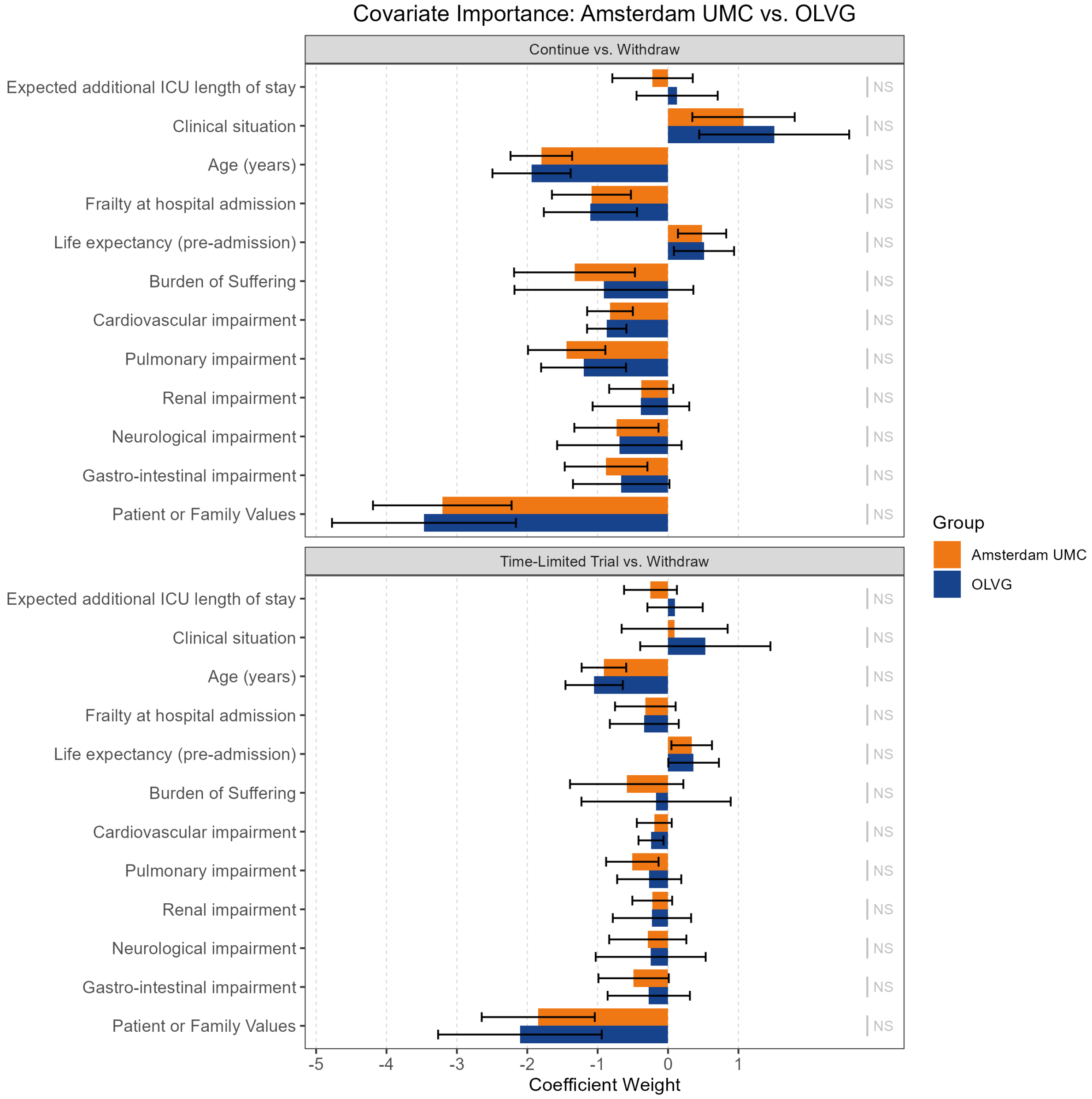


**Figure S3. Comparison of Covariate Importance**. Covariate importance is expressed as coefficient weights. Differences in coefficient weights were tested using the Wald test in a pooled interaction model of both groups. Error bars show 95% confidence intervals.

Abbreviations: *NS* not statistically significant. * $p < 0.05$. ** $p < 0.01$.
